# WikiPathways App for Cytoscape: Making biological pathways amenable to network analysis and visualization

**DOI:** 10.12688/f1000research.4254.2

**Published:** 2014-09-11

**Authors:** Martina Kutmon, Samad Lotia, Chris T Evelo, Alexander R Pico

**Affiliations:** 1 Department of Bioinformatics - BiGCaT, Maastricht University, Maastricht, 6229 ER, Netherlands; 2 Gladstone Institutes, San Francisco, CA 94158-2261, USA

## Abstract

In this paper we present the open-source WikiPathways app for Cytoscape ( http://apps.cytoscape.org/apps/wikipathways) that can be used to import biological pathways for data visualization and network analysis. WikiPathways is an open, collaborative biological pathway database that provides fully annotated pathway diagrams for manual download or through web services. The WikiPathways app allows users to load pathways in two different views: as an annotated pathway ideal for data visualization and as a simple network to perform computational analysis. An example pathway and dataset are used to demonstrate the functionality of the WikiPathways app and how they can be combined and used together with other apps. More than 3000 downloads in the first 12 months following its release in August 2013 highlight the importance and adoption of the app in the network biology field.

## Introduction

Pathways are commonly used as models for understanding biological processes. WikiPathways ^1^ is an open, collaborative, wiki-based website for the curation of biological pathways that are more than just images. WikiPathways provides easy-to-use drawing and annotation tools to capture identities, relationships, comments and literature references for each pathway element and interaction. Contributed pathways are displayed like articles at WikiPathways and can be downloaded manually or programmatically through web services. This opens the possibility for pathway information to be accessed by other software tools for data visualization, computational analysis and the interpretation of large-scale experimental data.

Utilizing WikiPathways web services, we developed an app for Cytoscape ^2^, a network visualization and analysis software platform. The app queries and imports pathways from WikiPathways within the Cytoscape environment. Cytoscape’s core concepts are networks (nodes and edges), tables (rows and columns) and styles, which map table values to the visual properties of networks. Cytoscape leverages a rich ecosystem of apps to provide additional domain-specific semantics and data types, as well as custom visualization and analysis capabilities. With the WikiPathways app, we implemented two ways to represent a pathway as a Cytoscape network. In the first way, pathways are loaded with the complete visual appearance of the original at WikiPathways, including graphical annotations and labels. Once in Cytoscape, experimental data can be loaded as tables and visually mapped onto these pathway-style networks to provide biological context. In the second way, pathways are loaded as simplified networks, focusing on the biological entities and their interactions without any of the graphical elements of the original pathway diagram. The basic network style is ideal for topological analyses, network merging and automatic layout.

In this paper we present the implementation and usage of the WikiPathways app for Cytoscape. By bringing pathways into Cytoscape using the WikiPathways app, it is possible to make full use of pathway models with custom visualizations and computational analyses.

## Implementation

The WikiPathways app was developed for Cytoscape 3, which introduced a completely new software architecture. The new architecture is built on top of Open Service Gateway Initiative (OSGi) ^3^, a software framework of pluggable modules and services. To be able to take advantage of the new architecture (Cytoscape API version 3.0.0), the predecessor to the WikiPathways app, the GPML Plugin, had to be rewritten.

### Pathway import

The WikiPathways app employs the new architecture of Cytoscape in two ways. First, the app exports a user interface that can query and import pathways from the WikiPathways web service. Thanks to the service architecture in Cytoscape, this interface is seamlessly incorporated into Cytoscape’s “Import from Public Databases” dialog. Second, the app provides an API for programmatic access to the WikiPathways web services and the GPML file importer. Other apps can use the API to make queries to the WikiPathways web services and import GPML files without having to bundle the WikiPathways app. When the WikiPathways app is loaded in Cytoscape, the app registers the implementation of its API with the OSGi module system. Other apps can then request the API implementation through OSGi.

### Visualization

The new architecture also posed new challenges that required us to innovate with respect to visual styles. The new architecture includes a revamped model to represent networks. This model decouples the network topology and table data from its visual style. Visual styles constitute Cytoscape’s view model. When a node or edge is created in the network model, its view object is only created after a triggering of an event. Cytoscape does this to avoid redrawing of the network canvas while an app is still in process of building the network. Indeed, as the WikiPathways app reads a GPML file, it creates a series of nodes and edges in a network to represent the pathway. During this process, the app needs to assign visual styles to the nodes and edges it creates. However, as new nodes and edges are being added to the network, their view objects do not exist yet, making it impossible to assign their visual styles. To address this issue, we created a class called **DelayedVizProp** that stores our desired visual styles for nodes and edges. Once the network has been fully built, the app tells Cytoscape to create the view objects for the new nodes and edges. After that, the app looks through the **DelayedVizProp** instances and assigns nodes and edges their desired visual style.

### Dependencies

The app relies on the PathVisio core library ^4^ to read GPML files. The PathVisio library is included in the app. In previous versions of Cytoscape, apps that included libraries often conflicted with each other. Users had to painstakingly uninstall conflicting apps for Cytoscape to become usable again. OSGi solves this problem by insulating Cytoscape modules and apps from each other. Due to OSGi’s architecture in Cytoscape 3, the integrated PathVisio library is hidden from other apps and modules in Cytoscape and cannot conflict with them.

The app also uses the Apache HTTP Client library to make HTTP requests to the WikiPathways REST server. We avoided the Java built-in HTTP client class ( **java.net.HttpURLConnection**), which is used frequently in Cytoscape and other apps. This class does not support cancellation. Proper cancellation is important for a responsive user interface. Users behind an interrupted internet connection should be able to back out of a WikiPathways request and return to Cytoscape. Each HTTP request is wrapped in a task, a unit of work in Cytoscape. When the user clicks cancel during the task execution, the app terminates the underlying HTTP request by calling the abort method in the Apache HTTP Client library.

## Results

**Figure 1.**
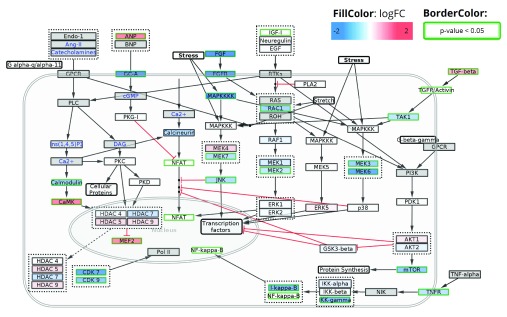
The Cardiac Hypertrophic Response pathway loaded as a pathway. LogFC values are visualized as node fill color with a color gradient from blue over white to red. Significant measurements (adjusted p-value < 0.05) are highlighted with a green border color. Elements in the pathway without a measurement are colored grey.

**Figure 2.**
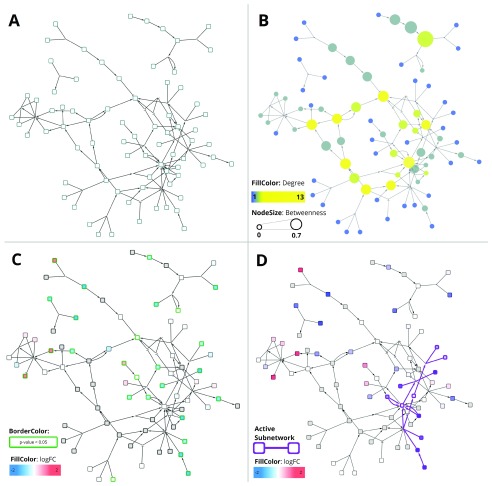
The Cardiac Hypertrophic Response pathway loaded as a network. ( **A**) The simple network does not contain graphical annotations of the pathway. ( **B**) NetworkAnalyzer was used to visualize node degree and betweenness of the nodes in the network to identify important hub nodes. ( **C**) The logFC of the example dataset is visualized as node fill color with a gradient (blue over white to red) and the adjusted p-value < 0.05 is highlighted with a green border color. ( **D**) jActiveModules finds active subnetworks (highlighted in purple) that are affected by varying gene expression.

The WikiPathways app in Cytoscape provides convenient access to the community-curated collection of biological pathways at WikiPathways. The functionality of the app is demonstrated here using the human Cardiac Hypertrophic Response pathway from WikiPathways ( http://wikipathways.org/instance/WP2795) combined with an unpublished RNA-seq dataset that reflects gene expression levels during differentiation of cardiac stem cells (see Dataset 1). The logFC from *timepoint 6 hrs vs control* is visualized on the pathway. The human Cardiac Hypertrophic Response pathway contains gene products and metabolites involved in the intracellular signal-transduction pathways that coordinate Cardiac Hypertrophic Response. As described above, the WikiPathways app allows users to load pathways in two different views, as an annotated pathway and as a simple network (see Figure 1 and Figure 2). The example dataset and pathway will be used to explain how both views can be used in Cytoscape.

When loaded as a pathway, the precise layout of elements is identical to its representation at WikiPathways. The graphical elements, like labels and shapes, are included in the model in Cytoscape. As a pathway diagram, the full representation of biological information is visually preserved, which is ideal for providing a meaningful context for data visualization. Figure 1 shows the Cardiac Hypertrophic Response pathway loaded as an annotated pathway in Cytoscape. The Entrez Gene identifiers in the pathway were mapped to Ensembl using another app called BridgeDb ^5^ ( http://apps.cytoscape.org/apps/bridgedb) to match the identifiers used in the example dataset. The cardiac stem cell tissue development expression data can then be loaded, integrated and visualized on the pathway nodes (c.f. Introduction to Cytoscape tutorial, http://opentutorials.cgl.ucsf.edu/index.php/Tutorial:Introduction\_to\_Cytoscape\_3.1-part2).

When loaded as a network, all graphical annotations are removed and redundant nodes in the pathway are merged into one unique node in the network. Groups and complex interactions are visualized as very small nodes and a forced directed layout is applied. As an abstracted network graph, the same molecular relationships in the pathways can be made available for network analysis and augmentation. Figure 2A shows the Cardiac Hypertrophic Response pathway loaded as a network in Cytoscape. This simple network structure enables researchers to use other Cytoscape features and apps to merge two pathways, apply different layouts to the network or extend the pathway, for example, with regulatory interactions (CyTargetLinker ^6^, http://apps.cytoscape.org/apps/cytargetlinker). It also enables users to investigate the topology of the network, like calculating degree and betweenness of the nodes with Cytoscape’s built-in NetworkAnalyzer tool to identify important hub nodes, see Figure 2B. Cytoscape also allows the visualization of experimental data in the network, as in Figure 2C which shows the cardiac stem cell tissue development expression data. There are several apps available for Cytoscape that provide methods that use experimental data to cluster nodes in the network (clusterMaker2, http://apps.cytoscape.org/apps/clustermaker2) or find subregions in the network affected by varying gene expression (jActiveModules, http://apps.cytoscape.org/apps/jactivemodules) as highlighted in Figure 2D.

Dataset studying differentiation of cardiac stem cells This is a subset of an unpublished RNA-seq dataset containing measurements for all genes in the selected Cardiac Hypertrophy Response pathway comparing “time point 6 hrs vs. control”. The dataset contains logFC, p-value and adjusted p-value measurements for every gene in the pathway.

## Conclusions

In this paper we presented the WikiPathways app for Cytoscape, which imports biological pathways as curated diagrams or as basic node-and-edge networks into Cytoscape. The process of transforming an arbitrary XML format like GPML into even a basic import format for Cytoscape is impractical without this dedicated app. The WikiPathways app thus provides researchers with a new, convenient method for accessing pathway information. Furthermore, as shown in the examples above, the app makes full use of the pathway models, allowing researchers to perform computational analyses and custom visualizations in conjunction with experimental data and network topology.

## Software availability

Software available from the Cytoscape App Store: http://apps.cytoscape.org/apps/wikipathways

Latest source code: https://github.com/wikipathways/cytoscape-wikipathways-app

Source code as at the time of publication: https://github.com/F1000Research/cytoscape-wikipathways-app

Archived source code as at the time of publication: http://dx.doi.org/10.5281/zenodo.10204 ^8^

License: Apache License, Version 2.0: http://www.apache.org/licenses/LICENSE-2.0.html

## Data availability

F1000Research: Dataset 1. Dataset studying differentiation of cardiac stem cells, 10.5256/f1000research.4254.d28415 ^7^

## Figures and Tables

**Supplementary Figure 1.**
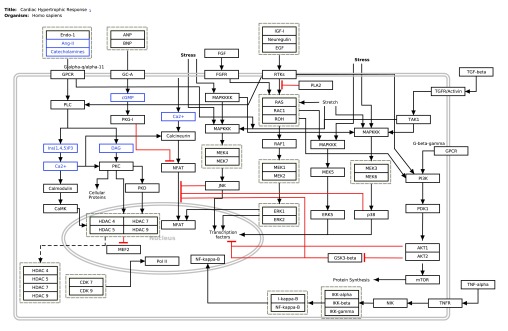
Human Cardiac Hypertrophic Response pathway from WikiPathways used in the manuscript: http://wikipathways.org/instance/WP2795_r76316.

**Supplementary Figure 2.**
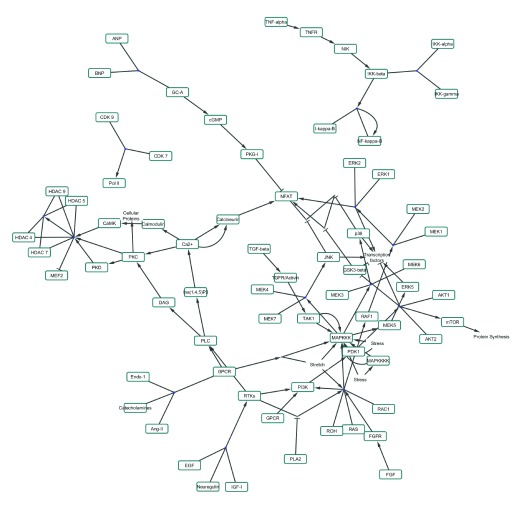
Figure 2A in the WikiPathways App paper including all graphical details, e.g. Labels.

**Supplementary Figure 3.**
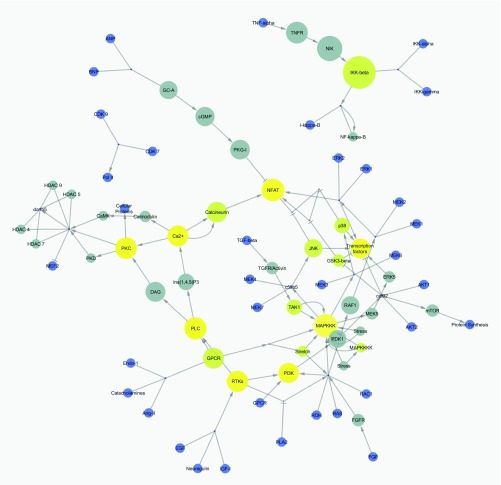
Figure 2B in the WikiPathways App paper including all graphical details, e.g. Labels.

**Supplementary Figure 4.**
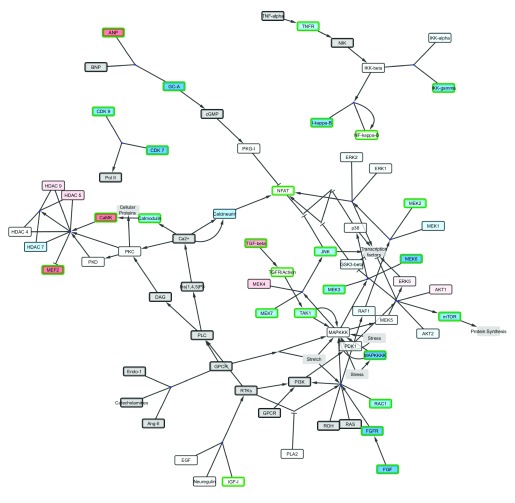
Figure 2C in the WikiPathways App paper including all graphical details, e.g. Labels.

**Supplementary Figure 5.**
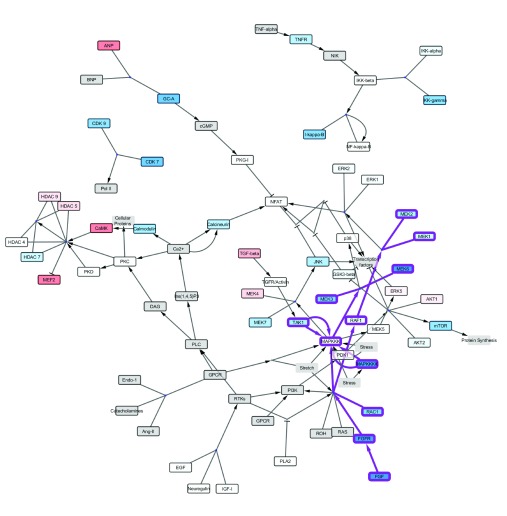
Figure 2D in the WikiPathways App paper including all graphical details, e.g. Labels.
