# Supplementary material for: WikiPathways App for Cytoscape: Making biological pathways amenable to network analysis and visualization
Source: F1000Res. 2014 Sep 11;3:152. Originally published 2014 Jul 1. [Version 2] doi: 10.12688/f1000research.4254.2 (PMC4168754; doi:10.12688/f1000research.4254.2)
Supplement: Peer review history

# Peer review: Referee response for version 1

**DOI:** 10.5256/f1000research.4555.r5523

**Published:** 2014-08-04

**Authors:** Ted Goldstein

**Affiliations:** 1 Department of Biomolecular Engineering, University of California Santa Cruz, Santa Cruz, CA, USA

You don't really make the point in the conclusions of the key findings of this excellent work which are:

1. It is now possible to do something quickly that before was tedious and therefore would not be done by anyone, except the most determined and experienced Cytoscape user.
2. It is now possible to do something that could not be done before because you needed to add new functionality.

I think it would be clearer that these are the main results if you add the following to the results section:

I suggest that you make a before and after video. The before video is the experience of using Cytoscape, manually downloading the data from Wikipathways,  failing to see the the nodes because it does not have the DelayedVizProp class etc.

Then the after video shows the full experience.

The elapsed time of the before video is something like 10 minute while the after video is something like 1 or 2 minutes. This will clearly demonstrate the improvement in the user experience.  Please report these time numbers in the text in the results section and possibly summarize the numbers in the abstract. 

Of course, also post these videos to both *F1000Research *and Youtube as they represent the primary data of your study.

I have read this submission. I believe that I have an appropriate level of expertise to confirm that it is of an acceptable scientific standard.

# Response

**Published:** 2014-08-07

**Authors:** Alexander Pico

**Affiliations:** The Gladstone Institutes, UCSF, USA

Dear Dr. Goldstein,

Thank you for the enthusiastic review and suggestions. We have drafted a new conclusions paragraph to include in the next revision to the article, which should be posted very soon. Coincidentally, we were gearing up to prepare a series of introductory videos for WikiPathways, each under 10 minutes long. We will certainly have one covering the use of the WikiPathways app for Cytoscape and will link/post it here as well. Given limited time and resources, we might not get around to producing the "before" video, but we will describe the process for contrast.  In fact, the "before" approach is not so clear. There are many routes to transforming an arbitrary XML like GPML into a input file for Cytoscape, ranging from the most basic SIF to the full xGMML formats. And for each format, one must make judgement calls and compromises, and ideally rely on someone with detailed knowledge of the source format. So, that approach could take anywhere from a day to a week of scripting depending on the desired accuracy and purpose. And, of course, it takes months of planning and programming (like we did) to get the general-purpose app result we arrived at.

Thanks again for highlighting these points and for your suggestions!

# Peer review: Referee response for version 1

**DOI:** 10.5256/f1000research.4555.r5286

**Published:** 2014-07-02

**Authors:** Christian A Grove

**Affiliations:** 1 Division of Biology and Biological Engineering, California Institute of Technology, Pasadena, CA, USA

This paper is well written, clear and to-the-point. The title is appropriate and the abstract provides sufficient clarity and detail. The descriptions of the development, usage, and purpose of the WikiPathways App for Cytoscape are sufficiently detailed and informative for a bioinformatician such as myself. I cannot speak to the sufficiency of the detail of code and programming descriptions, although it seems reasonably complete. I've tested the App as the authors have described and found it to be easy to install and use with the set of descriptions and instructions provided on the source website and in this paper. All datasets and source code are easily obtainable with the links provided by the authors.

I have found one small typo in the network/pathway which I have manually changed at the WikiPathways.org site: the original network referred to the node of a gene product as "PCL", which according to the cited reference and the position in the network is surely meant to represent "Phospholipase C" which has the canonical abbreviation of "PLC", in the citation for the network and in numerous previous studies. Unless I have made this observation in error, I would recommend changing this in the network and associated figures.

I have read this submission. I believe that I have an appropriate level of expertise to confirm that it is of an acceptable scientific standard.
